# Dissecting the neuronal vulnerability underpinning Alpers’ syndrome: a clinical and neuropathological study

**DOI:** 10.1111/bpa.12640

**Published:** 2018-10-09

**Authors:** Hannah Hayhurst, Maria‐Eleni Anagnostou, Helen J. Bogle, John P. Grady, Robert W. Taylor, Laurence A. Bindoff, Robert McFarland, Doug M. Turnbull, Nichola Z. Lax

**Affiliations:** ^1^ Wellcome Centre for Mitochondrial Research, Institute of Neuroscience, Newcastle University Newcastle upon Tyne NE2 4HH UK; ^2^ Department of Clinical Medicine University of Bergen Bergen Norway; ^3^ Department of Neurology Haukeland University Bergen Norway

**Keywords:** alpers’ syndrome, mitochondrial DNA, neurodegeneration, polymerase gamma, respiratory chain deficiency

## Abstract

Alpers’ syndrome is an early‐onset neurodegenerative disorder often caused by biallelic pathogenic variants in the gene encoding the catalytic subunit of polymerase‐gamma (*POLG*) which is essential for mitochondrial DNA (mtDNA) replication. Alpers’ syndrome is characterized by intractable epilepsy, developmental regression and liver failure which typically affects children aged 6 months–3 years. Although later onset variants are now recognized, they differ in that they are primarily an epileptic encephalopathy with ataxia. The disorder is progressive, without cure and inevitably leads to death from drug‐resistant status epilepticus, often with concomitant liver failure. Since our understanding of the mechanisms contributing the neurological features in Alpers’ syndrome is rudimentary, we performed a detailed and quantitative neuropathological study on 13 patients with clinically and histologically‐defined Alpers’ syndrome with ages ranging from 2 months to 18 years. Quantitative immunofluorescence showed severe respiratory chain deficiencies involving mitochondrial respiratory chain subunits of complex I and, to a lesser extent, complex IV in inhibitory interneurons and pyramidal neurons in the occipital cortex and in Purkinje cells of the cerebellum. Diminished densities of these neuronal populations were also observed. This study represents the largest cohort of post‐mortem brains from patients with clinically defined Alpers’ syndrome where we provide quantitative evidence of extensive complex I defects affecting interneurons and Purkinje cells for the first time. We believe interneuron and Purkinje cell pathology underpins the clinical development of seizures and ataxia seen in Alpers’ syndrome. This study also further highlights the extensive involvement of GABAergic neurons in mitochondrial disease.

**Abbreviations**
ANS: Ataxia neuropathy spectrum
CFV: Cresyl fast violet
CPEO: chronic progressive external ophthalmoplegia
COX: cytochrome *c* oxidase
COX1: cytochrome *c* oxidase subunit 1
COX4I2: cytochrome *c* oxidase subunit 4 isoform 2
EEG: electroencephalogram
FFPE: formalin‐fixed paraffin‐embedded
GAD65‐67: glutamic acid decarboxylase 65‐67
HS: hippocampal sclerosis
MEMSA: myoclonic epilepsy myopathy and sensory ataxia
MRI: magnetic resonance imaging
mtDNA: mitochondrial DNA
NDUFB8: NADH dehydrogenase [ubiquinone] 1 beta subcomplex subunit 8
NDUFA13: NADH dehydrogenase [ubiquinone] 1 alpha subcomplex 13
OD: optical density
POLG: polymerase‐gamma
ROI: region of interest
SDH: succinate dehydrogenase
TLE: temporal lobe epilepsy

## Introduction

Alpers’ syndrome, more correctly described as Alpers‐Huttenlocher syndrome, is a severe early‐onset neurodegenerative disorder characterized by a clinical triad of intractable epilepsy, psychomotor regression and liver failure 2. Other symptoms include developmental delay, failure to thrive, hypotonia, spasticity and ataxia. The exact prevalence of Alpers’ syndrome is unknown due to high mortality and early death but it is estimated to affect 1:100,000 births 38. A bimodal disease onset has been described with the first peak of onset affecting infants between the ages of 6 months and 3 years and a second peak, typified by an epileptic encephalopathy, during adolescence 16, 48. The disorder is progressive, without cure and is invariably fatal caused by status epilepticus or hepatic failure.

The typical clinical presentation of Alpers’ syndrome is sudden explosive onset of medically refractory seizures in an apparently healthy and normally developing child or young adult. Initially, seizures may manifest as focal motor seizures and/or myoclonus and often progress to status epilepticus or focal motor status, electroenecephalogram (EEG) typically demonstrates a focus within the occipital lobe 49. Concomitant symptoms may also include cortical blindness, vomiting, headaches, and hemianopia. Cortical and thalamic involvement are seen on magnetic resonance imaging (MRI) and are characterized by areas of high intensity signal on T2 and T2‐FLAIR weighted sequences corresponding to stroke‐like lesions 44, 46. Valproic acid was amongst a number of drugs used to treat the refractory epilepsy associated with Alpers’ syndrome until it became evident that it was precipitating liver failure 7.

More than 90% of cases of Alpers’ syndrome are thought to be due to autosomal recessive mutations in the nuclear‐encoded catalytic subunit of polymerase gamma (*POLG*) 7, 8, the sole polymerase responsible for replication and repair of mitochondrial DNA (mtDNA) 14. Mutations in *POLG* cause a spectrum of clinical phenotypes, including myoclonic epilepsy myopathy and sensory ataxia (MEMSA), ataxia neuropathy spectrum (ANS) and both dominant and recessive chronic progressive external ophthalmoplegia (CPEO) 50. *POLG* defects result in either mtDNA depletion or accumulation of mtDNA deletions, and in Alpers’ syndrome it is depletion of mtDNA which is responsible for the development of severe mitochondrial respiratory chain impairments, and consequent cellular dysfunction and cell death in brain and liver 4, 32, 46. Recent whole exome sequencing has identified other molecular genetic causes of Alpers’ syndrome, including pathogenic mutations in *FARS2* which encodes phenylalanyl‐tRNA synthetase 10, *NARS2* which encodes asparaginyl‐tRNA synthetase and *PARS2* which encodes prolyl‐tRNA synthetase 40, 41.

Neuropathological findings typically reveal symmetrical atrophy with laminar necrosis and neuronal degeneration affecting the cerebral cortex, hippocampi, olfactory bulbs and cerebellum 15, 16, 19. A recent neuropathological study of paediatric and adult POLG‐related disease revealed chronic neurodegeneration with areas of acute focal neuronal necrosis involving frontal, temporal and occipital cortices, and the hippocampus, thalamus and cerebellar cortex 46. Subsequent evidence has revealed focal lesions provide no evidence of ischaemia, suggesting this loss is secondary to neuronal ATP depletion caused by a combination of underlying respiratory chain deficiency and epileptic activity increasing neuronal energy demands 45.

In the cerebellum, mild Purkinje cell loss has previously been documented in Alpers’ syndrome 29, 31. Diffuse Purkinje cell loss has been described in a cohort of adult patients with *POLG* mutations, and reduced expression of respiratory chain complex I has been noted in remaining Purkinje cells of patients 8, 23. Numerous clinical and neuropathological studies reveal the occipital lobe as the main site of seizure genesis and a predominant area of neuronal degeneration in patients with Alpers’ syndrome 3, 11, 19, 29, 35. Extensive respiratory chain deficiency and interneuron loss has also been documented in occipital lobe tissue of patients with mitochondrial disease including three adult patients harboring recessively inherited *POLG* variants 22.

In the current study, we aimed to further delineate the pathogenesis of Alpers’ syndrome through a quantitative neuropathological investigation of a large series of post‐mortem brain tissues from thirteen patients with clinically‐defined Alpers’ syndrome. Our approach involved quantifying the extent of neuronal‐subtype specific cell loss in conjunction with assessing evidence of mitochondrial respiratory chain deficiencies, involving complexes I and IV, in occipital cortex and the cerebellum since these areas often show pronounced neurodegeneration.

## Materials and Methods

### Tissue material

Formalin‐fixed paraffin‐embedded (FFPE) brain tissue for the current study was acquired from the Newcastle Brain Tissue Resource, UK, BRAIN UK, Edinburgh Sudden Death Brain Bank, UK, University of Vienna, Austria, and University of Bergen, Norway. Where possible control cases were matched with our patients for age, sex, post‐mortem interval and length of tissue fixation (Supplementary Table 1). Not all brain regions were available for each patient and control and this is indicated by Supplementary Table 1. Ethical approval and full consent was granted for this work.

### Immunofluorescent identification of mitochondrial respiratory subunits in interneurons, pyramidal neurons and Purkinje cells

Immunofluorescent assays were developed to allow identification of subunits comprising complexes I and IV relative to a mitochondrial mass marker within specific neuronal populations. Since there are no reliable, commercially‐available antibodies raised against mtDNA‐encoded subunits of complex I, commonly affected nuclear‐encoded subunits of complex I (NADH dehydrogenase [ubiquinone] 1 beta subcomplex subunit 8 (NDUFB8) or NADH dehydrogenase [ubiquinone] 1 alpha subcomplex 13 (NDUFA13)) were selected in conjunction with a mtDNA‐encoded subunit of complex IV (cytochrome *c* oxidase subunit 1 (COX1)) and a marker of mitochondrial mass (porin or cytochrome *c* oxidase subunit 4 isoform 2 (COX4I2)). While there are no reliable histochemical methods to asses complex I activity in tissue cryosections, previous work has shown that complex I NDUFB8 and NDUFA13 subunits are incorporated at late stages of complex I assembly and are an essential part of functional enzymatic activity 5, 34. Downregulation of COX1 protein expression levels has been show to directly correlate to loss of COX activity upon cytochrome *c* oxidase (COX)/succinate dehydrogenase (SDH) histochemistry in tissue cryosections 28. Based on these studies, we infer that decreased protein levels can reliably be interpreted as reflecting enzymatic activity. We have optimized and validated these antibodies extensively within other studies of single muscle fibers 1, 36 and various brain‐specific cell types in tissue sections from patients with mitochondrial disease 8, 13, 22.

In occipital lobe tissue, these antibodies were used in conjunction with neuronal markers; anti‐SMI‐32P an antibody that recognizes non‐phosphorylated neurofilaments in pyramidal neurons and anti‐glutamic acid decarboxylase 65‐67 (GAD65‐67) an antibody that identifies GABAergic interneurons. The combinations of primary antibodies were selected on the basis of different IgG subtypes. Due to an overlap in IgG1 isotype for SMI‐32P and NDUFB8 two triple‐labeled assays were used: the first to identify complex I, mitochondria and pyramidal neurons and the second to identify complex IV, mitochondria and pyramidal neurons. These assays were applied to 8 patients and 4 controls were FFPE occipital lobe tissue was available. In the cerebellum, porin was used to define the Purkinje cell body due to their characteristic morphology therefore the assay did not utilize a neuronal marker; this was applied to 9 patients and 8 controls. All primary and secondary antibodies used are listed in Supplementary Table 2. A no primary antibody (NPA) section was included alongside patients and controls for each brain region.

Immunofluorescence was performed on 5 μm FFPE brain sections as previously described 12, 22. This briefly involved deparaffinization and rehydration of the sections followed by antigen retrieval and blocking in 10% goat serum for an hour. Primary antibodies were incubated overnight at 4°C, the following day and where applicable IgG subtype‐specific biotinylated goat anti‐mouse antibodies were applied to amplify either NDUFB8 or NDUFA13 antibody signal which has previously been shown to be weak. Sections were incubated for 2 h at 4°C, sections were washed with TBST and the appropriate IgG subtype‐specific secondary anti‐mouse or anti‐rabbit antibodies conjugated with Alexa Fluor 405, 488, 546 and 647 antibodies applied for 2 h at 4°C (see Supplementary Table 2). When possible the sections were incubated with Hoescht (Sigma Aldrich; diluted 1:1200 with TBST) for 20 minutes at room temperature to label nuclei. All sections were incubated in 0.3% Sudan Black for 10 minutes to eliminate any autofluorescence. This was followed by washing in distilled water and mounting in Prolong Gold (Life Technologies, UK).

### Confocal microscopy

Neurons were imaged using a confocal microscope (Nikon A1R, UK). Neurons were detected using an immersion oil ×60 objective with numerical aperture 1.4 by their SMI‐32P or GAD65‐67‐positive signal. At least 40 neurons were randomly sampled per case using an electronic zoom of ×3.09. Purkinje cells in the cerebellum were detected using a ×20 objective on the basis of their porin signal and imaged at the same magnification. Microscope and laser settings were maintained throughout image capture.

### Image processing and analysis

Neurons were identified and manually outlined according to the presence of either GAD65‐67 405 nm (for interneurons), SMI‐32P 647 nm (for pyramidal neurons) or porin 546 nm (for Purkinje cells) fluorescent signal using Volocity software (PerkinElmer); this defined the region of interest (ROI). For each neuron, the mean optical densities (OD) of each mitochondrial respiratory complex subunit (NDUFB8, NDUFA13 or COX1) and mitochondrial mass marker (porin or COX4I2) were measured within the defined ROI and subject to background correction from NPA section.

The control group was created by sampling equal numbers of neurons from each control subject and pooling the background corrected mean OD values. Box‐Cox transformation identified the log transformation as optimal to normalise the data. Linear regression of transformed NDUFB8 (NDUFB8^T^) data against transformed porin (porin^T^) data, transformed NDUFA13 (NDUFA13^T^) data against transformed COX4I2 (COX4I2^T^) data and transformed COX1 (COX1^T^) data against porin^T^ data were performed to ensure the residuals of the regression were normally distributed. NDUFB8^T^, NDUFA13^T^ and COX1^T^ values were then corrected for mitochondrial mass by dividing by the appropriate mitochondrial mass marker (porin^T^ or COX4I2^T^). In order to derive z‐scores for each pyramidal neuron, interneuron and Purkinje cell the mass‐corrected NDUFA13 (NDUFA13^Z^), NDUFB8 (NDUFB8^Z^) and COX1 (COX1^Z^) values were calculated based on the parameters (mean and standard deviation) of the control group. Neurons were classified based on standard deviation (SD) limits (for NDUFA13^Z^, NDUFB8^Z^ or COX1^Z^: overexpression if z > 2SD, normal if z > ‐2SD, low if z < ‐2SD, deficiency if z < ‐3SD and severe deficiency if z < ‐4SD) and are presented as percentages to give the proportion of cells within each SD limit.

### Statistical analysis

All statistical analyses were carried out using GraphPad Prism 7.0 (GraphPad Software, Inc., La Jolla, California). To determine statistical significant changes in complex I or IV protein expression in each patient relative to the control group, median z scores for each patient and control were compared using an unpaired *t*‐test. To determine changes in porin or COX4I2, the mean OD per neuron in patient and control tissues was compared using a Mann–Whitney U test. To ascertain a correlation between neuronal cell loss, complex I or IV deficiency and age at death, Spearman rank correlation coefficient was performed. A significant *P*‐value cut off was taken at *P ≤ 0.05, **P ≤ 0.01 and ***P ≤ 0.001.

### Neurohistopathological staining

Immunohistochemistry for identification of pyramidal neurons (anti‐SMI‐32P; Covance 1:6,000) and interneurons (anti‐GAD65‐67; Sigma 1:6,000) using a polymer detection kit and visualization with DAB as previously described 24. 20μm sections of FFPE cerebellar tissue were stained with cresyl fast violet (CFV) allowing identification of Purkinje cells.

### Two‐dimensional neuronal cell quantification

A two‐dimensional neuronal cell counting protocol was employed using a modified light microscope (Olympus BX51), motorized stage for automatic sampling, CCD colour video and stereology software (StereoInvestigator, MBF Bioscience, Williston, VT) as previously described 22, 23. This briefly involved outlining an area of at least 10μm^2^ along the cortical ribbon for the occipital cortex encompassing cellular layers I–VI at ×2 magnification. Within this area GAD65‐67‐positive interneurons and SMI‐32P‐positive pyramidal neurons in occipital lobe tissues were counted at ×20 magnification. Purkinje cell density was calculated by identifying the Purkinje cell layer using the closed contour function of StereoInvestigator, accounting for their linear arrangement. Purkinje cells with a visible nucleolus were counted within this, and density calculated. Neuronal cell densities were calculated as number per mm^2^. Since only one measurement was provided per patient and control case, and due to the low numbers in each group, it was not possible to perform statistical analysis of neuronal cell density data.

## Results

### Clinical features

In total thirteen patients with clinically‐determined Alpers’ syndrome were investigated and a diagnosis of Alpers’ syndrome was made on the basis of the presence of refractory seizures/status epilepticus, psychomotor regression and/or liver failure (Table 1). A bimodal onset of Alpers’ syndrome is apparent with patients 1–10 and 12 developing symptoms early in infancy while patients 11 and 13 developed symptoms in later childhood. Seizures (including status epilepticus, epilepsia partialis continua or generalized seizures) were a major feature in all thirteen patients, and the presenting symptom in patients 2, 3, 5, 7, 8 and 13. Symptoms preceding seizure onset often included headaches, vomiting and visual abnormalities (including visual hallucinations and visual blindness). Other symptoms included psychomotor regression (delayed motor development, loss of previously acquired skills, minimal responsiveness) and liver failure affecting six patients.

**Table 1:** Genetic and clinical details for patients included in the current study

Patient Number	Age at presentation	Age at death	Sex	Primary clinical features									EEG finding	CT findings	Molecular genetics	Affected sibling	Previously published
				Seizures/status epilepticus	Visual symptoms	Liver failure/abnormal LFTs	Hemianopia	Hypotonia/ataxia/hemiplegia	Drowsiness/rapid fatigue	Headache	Vomiting	Fever					
Patient 1	Not known	2.5 m	F	+													
Patient 2	2 m	5.5 m	M	+				+					Slow base rhythm, frontal delta waves, generalized spikes.	Symmetrical hydrocephalus.		Affected sister.	
Patient 3	1 m	6.5 m	F	+					+		+		Hypsarrhythmia				
Patient 4	7 m	7 m	M	+		+									p.Ala467Thr/Gly848Ser		[29]
Patient 5	4 m	13 m	M	+	+	+		+	+				Hypsarrhythmia				
Patient 6	11 m	13 m	M	+		+									p.Gly303Arg/Ala467Thr		[29]
Patient 7	11 m	14 m	F	+		+		+	+		+		Widespread irregular alpha and theta activity mixed with moderate delta activity.	Mild generalized cerebral atrophy.	p.Ala647Thr/Gly848Ser	None	
Patient 8	17 m	27 m	M	+	+	+	+	+	+		+	+	Deterioration	Atrophy in left posterior region.	p.Ala647Thr/Thr914Pro	Unaffected sister.	[20]
Patient 9	2 m	4 y	F	+	+						+						
Patient 10	6 m	7 y	F	+	+			+			+		Asymmetric dysrhythmia with dominant delta activity in in fronto‐parietal regions, slow wave spike variants in occipital lobes.		p.Thr748Ser/Thr748Ser		[12]
Patient 11	6 y	12.5 y	M	+	+			+		+	+		Diffusely slow activity.	Diffuse symmetrical cerebral atrophy and enlarged lateral ventricles.			
Patient 12	1 y	14 y	F	+												Affected sibling.	
Patient 13	16 y	18 y	F	+	+	+		+		+	+			Ventricular dilation.		Affected brother. p.Trp748Ser, p.Arg1096Cys	

Molecular genetic diagnosis was achieved for five patients and Sanger sequencing confirmed the presence of mutated *POLG* variants (Table 1). Of these five patients; two harbored p.Ala467Thr/p.Gly848Ser, one harbored p.Gly303Arg/p.Ala467Thr, one harbored p.Ala467Thr/p.Thr914Pro and the final patient harbored homozygous p.Trp748Ser/p.Trp748Ser variants. Unfortunately genetic diagnosis has not been possible for the remaining eight patients since they died prior to genetic testing for *POLG* defects and extraction of DNA from fixed tissues did not allow for successful PCR amplification for screening of the three common variants associated with *POLG*‐related disease.

Four patients harboring biallelic *POLG* mutations have been previously reported; the clinical, genetic and neuropathological findings described for patients 4 and 6 by Tzoulis and colleagues 46, the clinical course and muscle biochemistry reported for patient 8 by Morris and colleagues 30 and the clinical and neuropathological findings reported for patient 10 by Jellinger and colleagues 17.

### Gross neuropathological findings

The gross neuropathological findings from post‐mortem are summarized by Table 2. The most common findings were significant atrophy affecting the cerebral hemispheres and focal areas of cortical depressions corresponding to areas of cortical laminar dehiscence caused by neuronal necrosis. Typically the occipital, parietal and cerebellar cortices were affected by severe, focal pan‐necrotic lesions featuring profound neuronal cell loss and a strong astrocytic response. These findings are compatible with previous descriptions in the literature 15, 45, 46.

**Table 2:** Summary of the gross neuropathological findings in patients with Alpers’ syndrome

Patient	Brain weight (g)	External macroscopic neuropathological findings	Histological findings						
			Occipital lobe	Parietal lobe	Frontal lobe	Cerebellum	Basal ganglia	Thalamus	Hippocampus
Patient 1^1^					Preserved	Astrogliosis	Significant neuronal loss	Mild gliosis	
Patient 2^1^			Atrophic gyri and severe narrowing of the white matter	Severe atrophy of gyri, marked cell loss in layers II and III with spongiosis and astrogliosis		Atrophy of the cerebellar lobes, mild to moderate purkinje cell loss	Marked gliosis in putamen and internal capsule, globus pallidus well‐preserved		
Patient 3^1^						Mild loss of Purkinje cells, Bergmann glia	Neuron loss and astrogliosis in the caudate nucleus, globus pallidus and putamen well preserved	Marked loss of neurons, astrogliosis	
Patient 4			Areas of focal neuronal necrosis			Mild neuronal loss	Caudate, putamen and globus pallidus are well preserved	Moderate pathology	Focal cell loss from CA1
Patient 5^1^			Atrophic gyri, gliosis	Atrophic gyri, gliosis	Narrowing of the cortical ribbon with areas of neuronal loss and ischaemia	Astrogliosis in the dentate nucleus	Caudate, putamen, and globus pallidus are well preserved	Preserved	Atrophic hippocampal formation
Patient 6			Areas of focal neuronal necrosis			No neuronal loss			Focal cell loss from CA1
Patient 7	860	Brain appears oedematous.	Mild spongiform changes	Mild spongiform changes in superficial layers, irregular surface of the molecular layer	Preserved	Marked loss of Purkinje cells, focal folial abnormalities in the vermis and granule cell diminution			Preserved
Patient 8	1000	Cerebral hemispheres reveal bilateral frontal lobe abnormality. There is a 20 × 15 mm lesion affecting the left and right occipital lobe	Prominent degeneration of superficial cortex, predominantly involving layer 2, 3 and 4 in gyri, but in sulci affecting 90% of neurons. Astrogliosis and spongiform change characterises layers 2 and 3	Atrophic gyri, loss of neurons and neuropil degeneration		Marked loss of Purkinje cells, and neuronal depletion from dentate nucleus	Caudate, putamen, and globus pallidus are well preserved	Ventrolateral nucleus and ventromedial nucleus are affected by neuronal shrinkage and mild spongiform change	Extensive pyramidal neuron loss and loss of dentate granule neurons is almost complete
Patient 9	563.1	Marked atrophy of cerebral hemispheres, most severe in occipital poles, and the cortical ribbon is thin throughout. Marked ventricular dilatation	Marked thinning of the cortical ribbon, subtotal neuron loss, accompanied by astrogliosis and secondary changes in white matter	Marked neuronal loss	Marked neuronal loss	Marked loss of granule cells, moderate loss of Purkinje cells and axonal torpedoes	Caudate, putamen and globus pallidus well preserved	Severe neuronal cell loss and astrogliosis	Preserved
Patient 10		Severe diffuse atrophy with marked internal and external hydrocephalus. Reduction in the width of the cerebral cortex associated with shrinkage of the cerebral white matter, while the cerebellum is preserved	Widespread spongy degeneration featuring neuron loss and glial proliferation. White matter changes throughout	Widespread spongy degeneration featuring neuron loss and glial proliferation. White matter changes throughout	Widespread spongy degeneration featuring neuron loss and glial proliferation. White matter changes throughout	Loss of Purkinje cells and proliferation of Bergmann glia	Atrophic	Spongy lesions accompanied by neuronal cell loss and astrogliosis	Preserved
Patient 11	1057	Occipital lobes are slightly atrophied, and cerebellar hemispheres atrophied	Widespread cortical necrosis typically affecting gyral crests, and extensive white matter damage	Neuronal loss, astrogliosis	Neuronal loss, astrocytosis	Patchy loss of Purkinje cells and some patchy Bergmann gliosis	Cell loss from putamen with myelin pallor affecting axons	Small foci of degeneration consisting of neuronal cell loss and axonal degeneration	
Patient 12	264	Brain is severely atrophic. All cerebral hemispheres gyri are thin at 3–4 mm in diameter	Most extensive area of atrophy, almost total loss of cortical neurons	Marked atrophy of the cortex and underlying white matter. Almost complete loss of cortical neurons	Marked atrophy of the cortex and underlying white matter. Almost complete loss of cortical neuons	Cerebellar folia are atrophic	Caudate, putamen, and globus pallidus are well preserved	Shrunken and gliotic	
Patient 13	1098	Marked atrophy of cerebral hemispheres. There is a 1cm x 6mm lesion affecting the lateral surface of the right frontal lobe. Cortical lesion evident in cerebellar hemispheres	Small areas of ischemic‐like destruction with astrocytosis	Extensive ischemic‐like damage, areas of total cortical destruction with astrocytosis	Small areas of ischemic‐like destruction with astrocytosis	Marked loss of Purkinje cells and areas of focal neuronal necrosis	Caudate, putamen and globus pallidus are well preserved	Depleted neuronal population density with marked astrocytic response	Presence of ischemic neurons in CA1

1 indicates that there was only limited neuropathological information for these patients since they are historical cases.

### Respiratory chain deficiency in interneurons and pyramidal neurons

Quantitative quadruple‐labeled immunofluorescence showed co‐localization of NDUFB8, COX1 and porin within control interneurons and pyramidal neurons. In both the occipital lobe and cerebellum, patient neurons showed a significantly higher mean OD of porin per cell than control neurons (Mann–Whitney U test, *P* < 0.0001) suggesting that mitochondrial mass was much higher in patient neurons.

**Figure 1:**
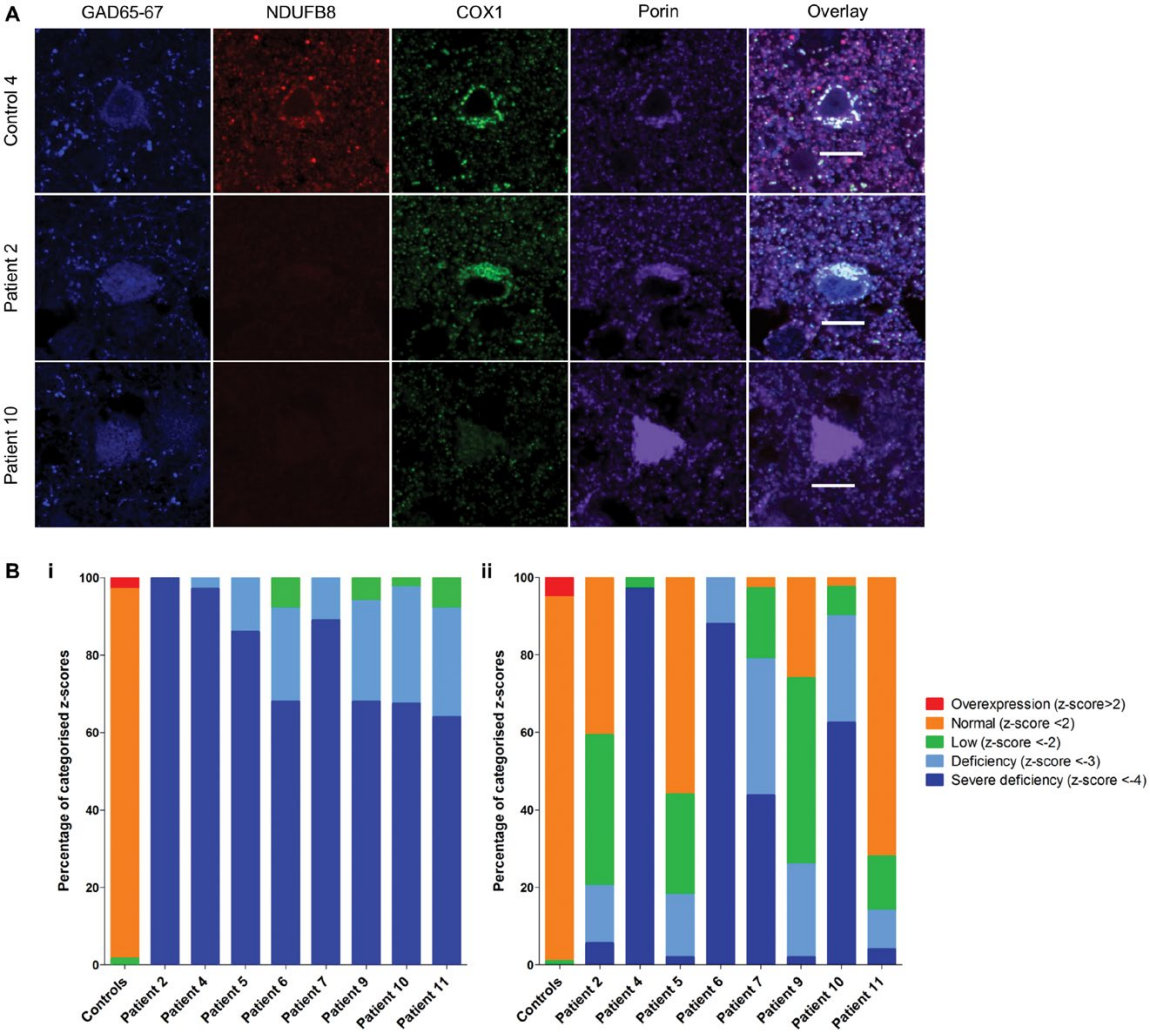
Downregulation of NDUFB8 and COX1 expression levels in patient GAD65‐67‐positive interneurons in occipital cortex. Representative images reveal downregulation of NDUFB8 and COXI expression levels relative to mitochondrial mass marker porin in patient inhibitory interneurons **A.** Scale bar = 10 microns. Quantitative analysis reveals the mitochondrial respiratory chain expression profiles for each patient and confirms higher percentage levels of NDUFB8 (unpaired *t*‐test, *P *< 0.0001) **B.i**. and COXI (unpaired *t*‐test, *P* < 0.0005) **B.ii.** deficiencies in interneurons.

Interneurons in the occipital cortex of patients exhibited significant downregulation of NDUFB8 (unpaired *t*‐test, *P* < 0.0001) and COX1 (unpaired *t*‐test, *P* < 0.0005) protein expression relative to mitochondrial mass marker porin (Figure 1A). In all patients a high proportion of interneurons demonstrated severe complex I deficiency (Figure 1Bi) while complex IV deficiency was more variable, with only patients 4 (p.Ala467Thr/p.Gly848Ser), 6 (p.Gly303Arg/p.Ala467Thr), 7 (pAla467Thr/p.Gly848Ser) and 10 (p.Thr748Ser/p.Thr748Ser) demonstrating severe complex IV deficiency in comparison to other patients (Figure 1Bii). All patient interneurons expressed complex I subunit NDUFB8 below the normal limits indicating that all interneurons exhibited severely deficient, deficient and low levels of NDUFB8 expression. While for complex IV subunit COX1 only two patients; patient 4 (p.Ala467Thr/p.Gly848Ser) and patient 6 (p.Gly303Arg/p.Ala467Thr), revealed downregulation of COX1 within all interneurons.

**Figure 2:**
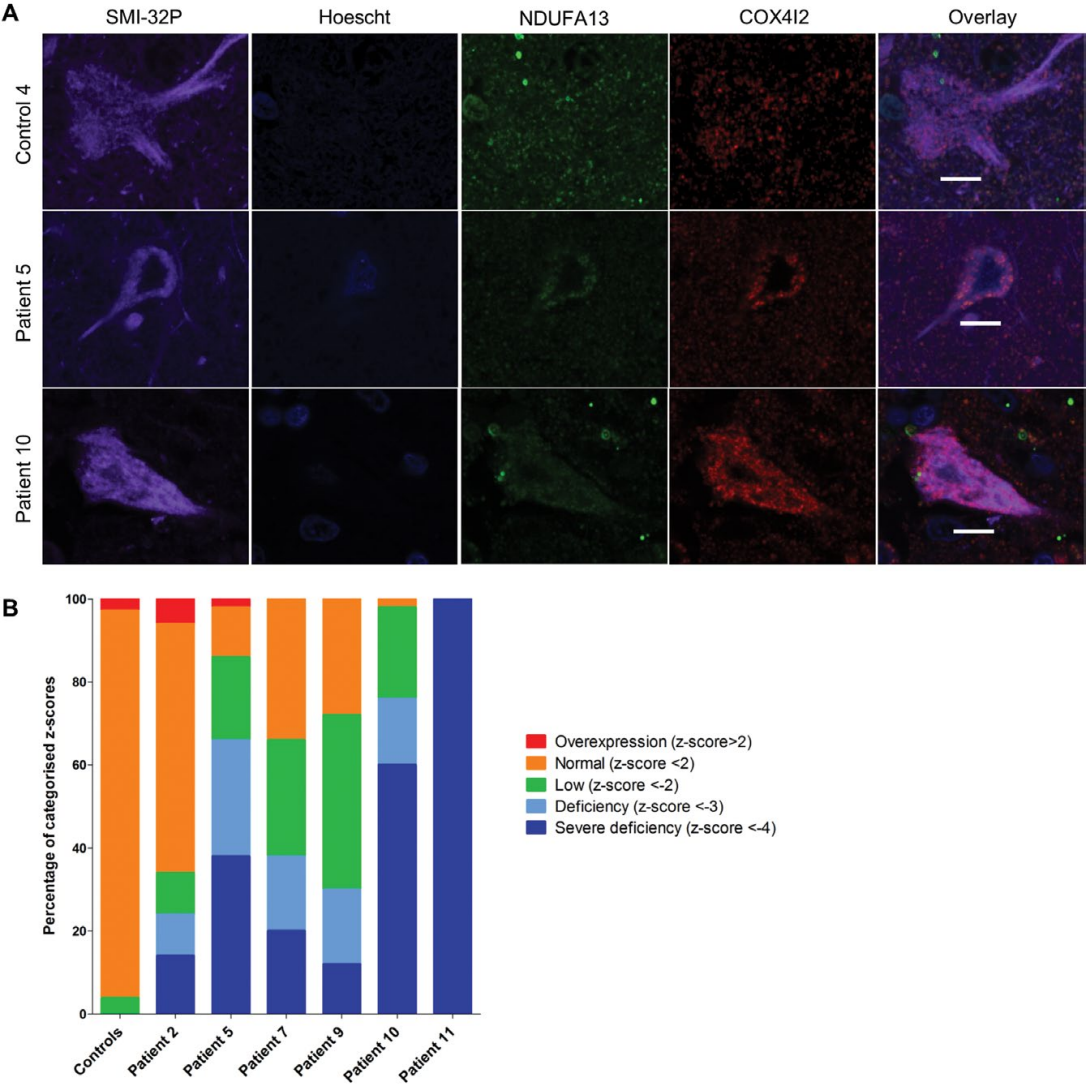
Downregulation of NDUFA13 expression levels in patient SMI‐32P‐positive pyramidal neurons within the occipital cortex. Representative images showing downregulation of complex I subunit NDUFA13 expression levels relative to complex IV subunit COX4I2 in pyramidal neurons from patients with Alpers’ syndrome **A.** Scale bar = 10 microns. Quantitative analysis reveals the mitochondrial respiratory chain expression profiles for each patient and confirms varying percentages of NDUFA13 deficiency in SMI‐32P‐positive pyramidal neurons (unpaired *t*‐test, *P* = 0.0358) **B**.

**Figure 3:**
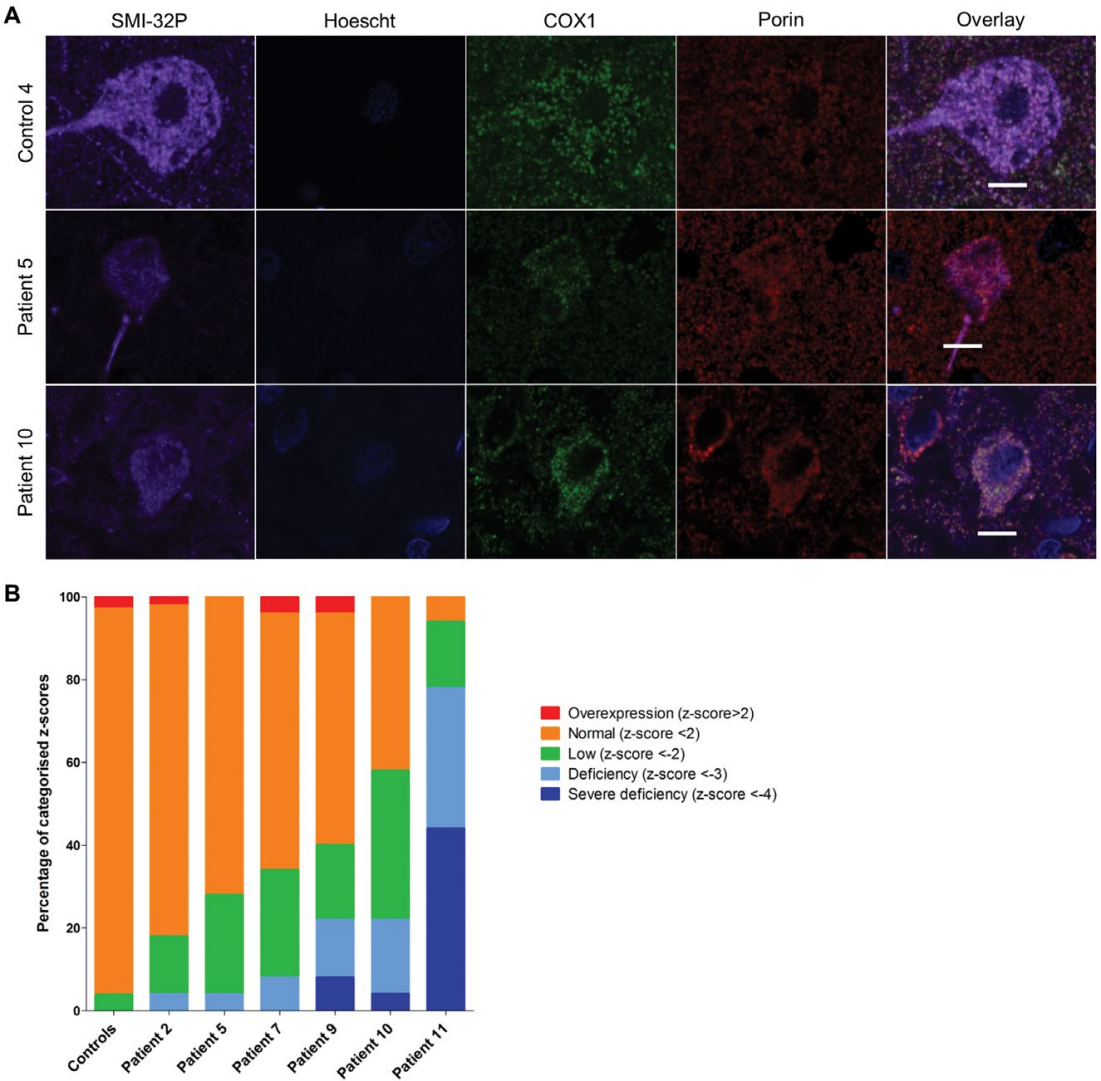
Downregulation of COX1 expression levels in patient SMI‐32P‐positive pyramidal neurons within the occipital cortex. Representative images showing downregulation of complex IV subunit COXI relative to mitochondrial mass marker porin in pyramidal neurons from patients with Alpers’ syndrome **A**. Scale bar = 10 microns. Quantification of protein expression in pyramidal neurons shows varying percentages of complex IV deficiency (unpaired *t*‐test, *P* < 0.0063) **B**.

Patient pyramidal neurons in the occipital cortex also demonstrated marked deficiency of NDUFA13 (unpaired *t*‐test, *P* = 0.0358; Figure 2A,B). However, this is likely an underestimation of complex I deficiency since comparison of COX4I2 OD (which is used as a mitochondrial mass marker in this assay) in patient and control neurons revealed a lower density of COX4I2 expression in neurons implying a loss of this protein in patient neurons. Significant downregulation of COX1 expression levels in patient pyramidal neurons was also observed (unpaired *t*‐test, *P* < 0.0063; Figure 3A,B). All patients showed high percentages of both complex I‐deficient and severely deficient neurons (Figure 2B). Patient 11 showed severe complex I deficiency in all neurons examined. Patient neurons showed milder complex IV deficiencies, with the most extensive deficiency being seen in patient 11 (Figure 3B).

### Respiratory chain deficient Purkinje cells of the cerebellum

**Figure 4:**
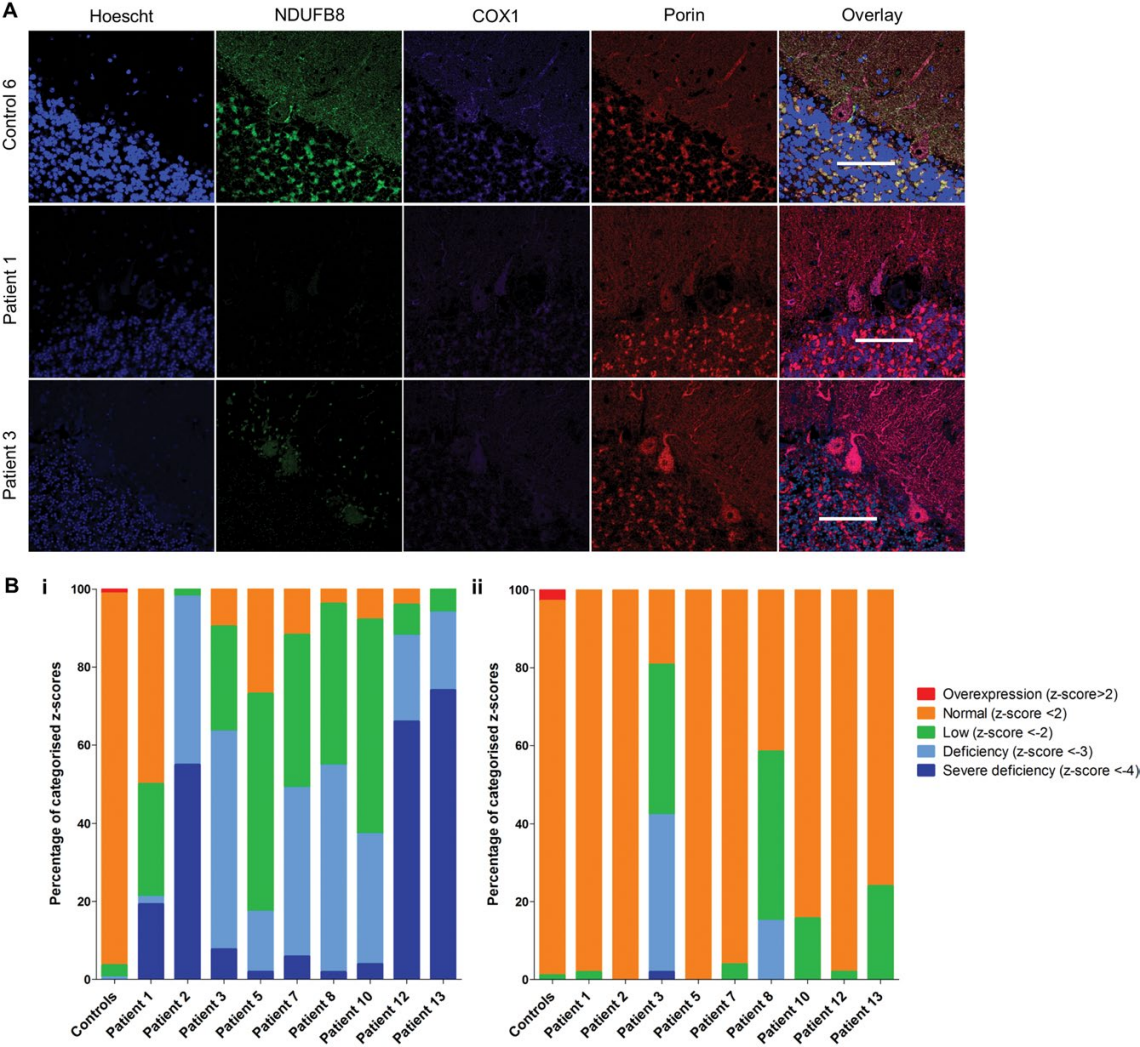
Downregulation of NDUFB8 and COX1 expression levels in patient Purkinje cells in the cerebellum. Representative images of immunofluorescent labeling of respiratory chain proteins NDUFB8, COXI and porin in Purkinje cells provide evidence of reduced NDUFB8 and COX1 expression despite intact mitochondria density (porin) in patient tissues **A**. Scale bar = 100 microns. Quantitation of the protein abundance provides mitochondrial respiratory chain profiles for each individual patient given as the percentage of complex I (NDUFB8; **B.i**, unpaired *t*‐test, *P* = 0.0002) and IV (COX1; **B.ii**, unpaired *t*‐test, *P* < 0.0064) expression in Purkinje cells.

The Purkinje cells in cerebellar cortex of patients exhibited decreased respiratory chain protein expression for both complexes I and IV (Figure 4). All patient Purkinje cells demonstrated a significant downregulation of complex I relative to control subjects (unpaired *t*‐test, *P* = 0.0002) with patients 1, 2, 12 and 13 exhibiting the highest levels of complex I deficiency in Purkinje cells. Complex IV expression was variable, and comparison of patient vs. control median z values revealed a significant downregulation in the patient cohort (unpaired *t*‐test, *P* < 0.0064), although only patients 3 and 8 (p.Ala467Thr/p.Thr914Pro) revealed neurons with complex IV deficiency.

### Neuronal loss in Alpers’ syndrome

**Figure 5:**
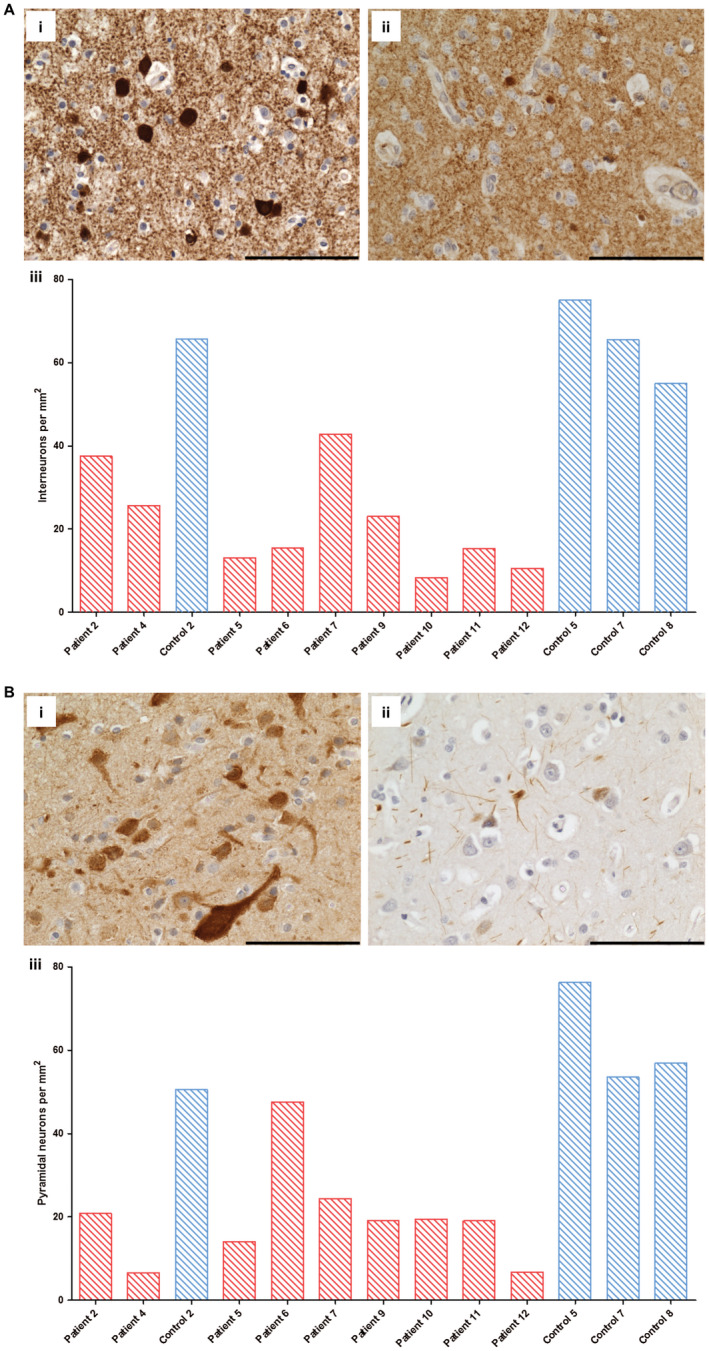
Decreased numbers of cortical inhibitory interneuron and pyramidal neurons in occipital cortex from patients with Alpers’ syndrome relative to control subjects. Immunohistochemical staining reveals GAD65‐67‐positive inhibitory interneurons in control (A. i; control 7) and fewer GAD65‐67‐positive interneurons in occipital cortex tissue from patients with Alpers’ syndrome (**A.ii**; patient 7). Scale bar = 100 microns. Quantification of GAD65‐67 cell density confirms a reduction in GABAergic interneuron density in Alpers’ syndrome (**A. iii**); patient = red bars, control = blue bars). Immunohistochemical staining reveals SMI‐32P‐positive pyramidal neurons in control (**A. i**; control 5) and reduced immunoreactivity for SMI‐32P and fewer SMI‐32P‐positive cells in Alpers’ syndrome (**B. ii**; patient 5). Scale bar = 100 microns. Quantification provides evidence of decreased pyramidal cell density in Alpers’ syndrome (**B. iii**; red bars = patients, blue bars = controls).

The consequence of respiratory chain deficiency was investigated by quantification of GABAergic and pyramidal neuronal cell densities in the occipital cortex and Purkinje cell density in the cerebellar cortex. The grey matter cortical ribbon demonstrated atrophy, with laminar dehiscence observed in patients 4 (p.Ala467Thr/p.Gly848Ser), 6 (p.Gly303Arg/p.Ala467Thr), 8 (p.Ala467Thr/p.Thr914Pro), 9, 11–13, characterized by neuronal dropout and spongy changes affecting all cortical layers. To understand the pattern of neuronal dropout, quantification of interneurons and pyramidal neuronal cell densities was performed. Quantification revealed reduced densities of both interneurons (Figure 5A) and pyramidal neurons (Figure 5B) throughout patient cortical layers relative to controls suggestive of interneuron and pyramidal neuron loss. Neuronal loss did not correlate with either complex I or complex IV deficiency in interneurons or pyramidal neurons, nor did it correlate with age (Spearman rank correlation coefficient).

**Figure 6:**
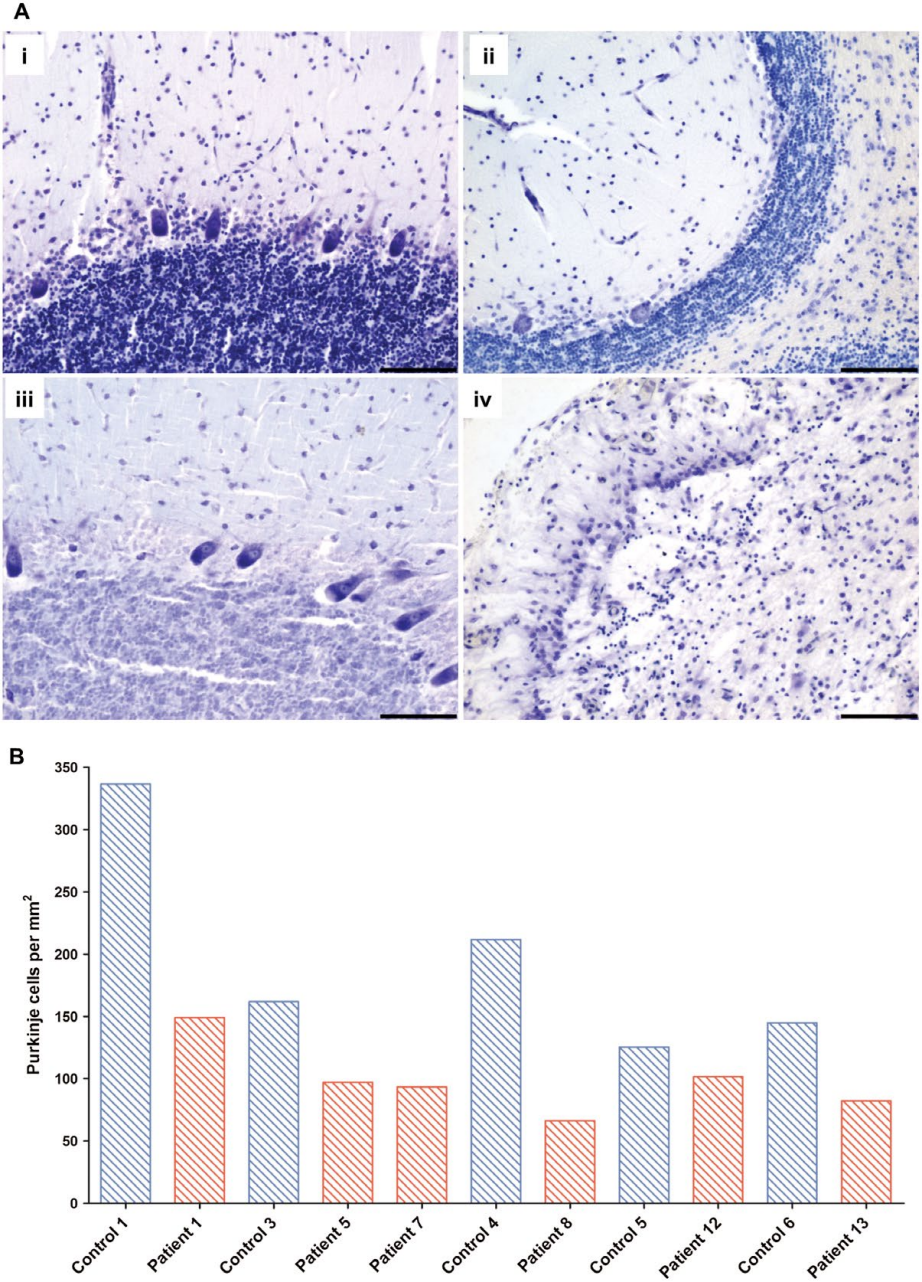
Purkinje cell density is reduced in patients with Alpers’ syndrome. Cresyl fast violet staining reveals the architecture of the cerebellar cortex in control (**Ai**; control 3) and shows Purkinje cell dropout in a patient with Alpers’ syndrome (**Aii**; patient 1). A comparison of age‐match control (**Aiii**: control 6) with an 18 year old patient with Alpers’ syndrome due to POLG mutations reveals a focal necrotic lesion featuring profound Purkinje cell and granule cell loss and destruction of the neuropil (**Aiv**; patient 13). Scale bar = 100 microns. Quantification of Purkinje cell density (B; red bars = patients, blue bars = controls) confirms reduced density in patients with Alpers’ syndrome.

The cerebellar cortex was atrophic in patients relative to controls, with a fewer Purkinje cells observed in the majority of cases (Figure 6Aii). An area of focal neuronal necrosis, consistent with a stroke‐like lesion, was seen in patient 13 (Figure 6Aiv). In other patients, Purkinje cells were selectively affected, with no apparent loss of granular cells or cells in the molecular layer on visual assessment. Quantification of Purkinje cell density revealed lower densities in patient cerebellum (Figure 6B) relative to controls. Purkinje cell loss did not correlate with age or with complex I or IV deficiency in surviving Purkinje cells (Spearman rank correlation coefficient).

## Discussion

Epilepsy, developmental regression and liver involvement are the classical features of Alpers’ syndrome. Due to the fulminant epilepsy, this syndrome is associated with poor prognosis and a rapidly progressive disease course. In the current study, epilepsy was a salient feature in all patients, and the presenting symptom in six. Although epilepsy is common in Alpers’ syndrome, the mechanisms underlying seizure generation and maintenance remain unclear. Using a novel quantitative approach, we investigated the development of neuronal respiratory chain deficiency and cell loss in a large cohort of patients with clinically defined Alpers’ syndrome. In particular, we focused on potential mechanisms contributing to seizures. We showed that occipital cortex GABAergic interneurons and pyramidal neurons, as well as Purkinje cells in the cerebellum, exhibited extensive respiratory chain defects as evidenced by significant downregulation of complex I subunit proteins and milder downregulation of complex IV subunit proteins. This was despite evidence of maintained or increased mitochondrial mass which could represent a compensatory response. Although loss of interneurons and pyramidal cells was similar, the magnitude of respiratory chain deficiency was more pronounced in interneurons raising the possibility that the loss of inhibitory neural networks plays a role in the seizure development in these patients.

A major limitation of this study is the absence of a confirmed genetic diagnosis for eight of our patients due a lack of availability of suitable tissues for molecular genetic testing. Unfortunately many of these cases are historical and precede the development of sequencing technologies to identify the causative genes for Alpers’ syndrome. Our criteria for inclusion of patient tissues was dependent on a clinical disease course and histopathological changes consistent with Alpers’ syndrome.

### Occipital lobe vulnerability in Alpers’ syndrome

The occipital lobe appears to be the main site for seizure genesis in Alpers’ syndrome and visual disturbances are also frequently described in both paediatric and adult patients harboring *POLG* mutations. Such visual phenomena, include flickering light, hallucinations, or loss of vision 11 and similar symptoms were described in seven of our patients. In this study, we confirm pathology affecting the occipital cortex with evidence of cortical atrophy, microvacuolation, and areas of focal neuronal necrosis in seven out of nine patients. We for the first time demonstrated quantitative evidence of extensive involvement of cortical interneurons in patients with Alpers’ syndrome. This study supports a vulnerability of GABAergic interneurons to mtDNA defects in patients with mitochondrial disease and is in agreement with a recent study revealing similar findings in a cohort of adult patients with mitochondrial disease 22. Unfortunately due to limited tissue availability, it was not possible to ascertain whether the severity and type of pathological changes were restricted to the occipital lobes and it will be important to extend these investigations to other cortical regions in Alpers’ syndrome in future studies.

### Cerebellar vulnerability in Alpers’ syndrome

In addition to the clear occipital involvement, we show a reduction in Purkinje cell density and respiratory chain defects, affecting subunits of complexes I and IV, in Alpers’ syndrome. This is a novel albeit unsurprising finding given ataxia is common in both paediatric and adult patients with this syndrome. Reduced Purkinje cell density combined with compromised respiratory chain protein expression has previously been documented in adult patients with mitochondrial disease, including those with *POLG* mutations 8, 23.

Cerebellar atrophy and Purkinje cell loss are common histological changes in patients with epilepsy 9, 37 and many develop ataxia 51. The specific mechanisms underpinning this pathology are unknown, however it could be attributed to seizure activity, particular status epilepticus 42 or due to toxicity of anti‐convulsants, such as phenytoin 9, 25, 26, 33. It is challenging to determine the precise mechanism by which the cerebellum is affected since both mechanisms may act synergistically. Unfortunately, we are unable to comment on phenytoin use in our patient cohort due to the scarcity of clinical information, however given the short‐nature of the disease, and extensive respiratory chain deficiency in remaining Purkinje cells we think it unlikely that the pathology is primarily caused by anti‐convulsant toxicity.

### Neuron‐specific pathology

Our data show GABAergic interneurons and pyramidal neurons of the occipital cortex, and Purkinje cells in the cerebellum exhibit extensive respiratory chain defects with significant downregulation of complex I subunit proteins coupled with milder downregulation of complex IV subunit proteins despite evidence of maintained or increased mitochondrial mass. These findings provide further support for the hypothesis that focal neuronal loss seen in *POLG*‐related stroke‐like lesions is a consequence of intrinsic neuronal energy failure rather than ischaemia 45. The consequence of the complex I deficiency within neurons we report in Alpers’ syndrome is not well understood, however the findings here support suggestions that complex I deficiency could be a contributory factor in the development of epilepsy 20.

Quantitative analysis of neuronal density showed significantly reduced densities of interneurons and pyramidal neurons in the occipital lobe, and reduced Purkinje cell density in the cerebellum in patients relative to controls. Previous studies have highlighted areas of focal cortical necrosis in the pathogenesis of Alpers’ syndrome; a finding observed in seven patients in this study 46. In the remaining six patients, neuronal loss appeared widespread throughout the occipital and cerebellar cortices. This could suggest two independent mechanisms of neurodegeneration are contributing to the pathology observed in Alpers’ syndrome.

Our findings of severe respiratory chain defects and loss of interneurons in the occipital lobes of our patients are novel and potentially offer the first clue to what may be driving the epilepsy in this disease. Epilepsy often heralds the rapid deterioration in patients with Alpers’ syndrome, and it was the only common symptom in all 13 patients in our cohort. It is striking, therefore, that patients 2, 4, 5 and 7 exhibited deficiency of complex I in all surviving occipital interneurons examined. Further, while neuropathological studies are limited by the fact that tissue demonstrates end‐stage disease, it is remarkable that in all patients in whom occipital lobe tissue was studied, there were no normal interneurons remaining. Recent electrophysiological studies have revealed a specific vulnerability of fast‐spiking interneurons to mitochondrial respiratory chain inhibitors, rotenone and potassium cyanide, which function to block complex I and IV activity 18, 47. Since interneurons often function as modulators of cortical neuron activity, the loss of any inhibitory activity could potentially lead to a lower seizure threshold and increase the probability of neuronal network becoming hyperexcitable. Indeed a specific pattern of inhibitory interneuron loss is reported in other epileptic disorders, such as temporal lobe epilepsy (TLE)/hippocampal sclerosis (HS), and experimental models of seizures and these alterations may contribute to sustained imbalances in excitatory/inhibitory activity which might perpetuate seizure activity in the brain 21, 27. Qualitative and quantitative alterations in specific inhibitory interneuron populations in TLE/HS tend to involve those expressing calcium binding proteins such as calbindin, calretinin and parvalbumin 6, 39, 43 and this specific vulnerability of interneurons should be explored in the context of Alpers’ syndrome.

Alpers’ syndrome is a rapidly progressive, incurable neurodegenerative disease and patients often die before 3 years of age. We present a detailed neuropathological characterization of post‐mortem tissue from thirteen patients that provides quantitative evidence of extensive complex I defects affecting interneurons and Purkinje cells. This is the most complete study to date and details the role of interneurons and how cell loss in both occipital lobes and cerebellum contribute to the pathogenesis of the seizures and ataxia.

## Conflict of interest

The authors declare no conflict of interest.

## Ethical approval

Newcastle and North Tyneside Local Research Ethics Committee (LREC2002/205) approved this study, and full consent for brain tissue retention and research was obtained.

## Supporting information

Table S1. Neuropathological details for patient and control tissues used in the current study.
Table S2. Primary and secondary antibodies used in this study.
