# Supplementary material for: Dissecting the neuronal vulnerability underpinning Alpers’ syndrome: a clinical and neuropathological study
Source: Brain Pathol. 2018 Oct 9;29(1):97–113. doi: 10.1111/bpa.12640 (PMC7379503; doi:10.1111/bpa.12640)
Supplement: Supplementary file 1 — Table S1. Neuropathological details for patient and control tissues used in the current study. Table S2. Primary and secondary antibodies used in this study. [file BPA-29-97-s001.docx]

**Supplementary Tables**

**Supplementary Table 1 – Neuropathological details for patient and control tissues used in the current study.**

COD = cause of death, PMI = post-mortem interval, N/A = data not available.

| **Patient Number** | **Source** | **Age at death** | **Sex** | **COD** | **PMI (hours)** | **Duration of fixation** | **Brain regions available** |
| --- | --- | --- | --- | --- | --- | --- | --- |
| Patient 1 | Vienna | 2.5m | F | Unknown | Unknown | Unknown | Cerebellum |
| Patient 2 | Vienna | 5.5m | M | Cardiac and respiratory failure | Unknown | Unknown | Cerebellum, occipital |
| Patient 3 | Vienna | 6.5m | F | Respiratory failure | Unknown | Unknown | Cerebellum |
| Patient 4 | Norway | 7m | M | Stroke-like episodes | Unknown | Unknown | Occipital |
| Patient 5 | Vienna | 13m | M | Pneumonia | Unknown | Unknown | Cerebellum, occipital |
| Patient 6 | Norway | 13m | M | Stroke-like episodes | Unknown | Unknown | Occipital |
| Patient 7 | NBTR | 14m | F | Hepatic failure | 12 | 1m | Cerebellum, occipital |
| Patient 8 | NBTR | 27m | M | Pulmonary haemorrhage and respiratory failure | 12 | 5w | Cerebellum |
| Patient 9 | Bristol | 4y | F | Broncho-pneumonia | 24 | Unknown | Occipital |
| Patient 10 | Vienna | 7y | F | Unknown | Unknown | Unknown | Cerebellum, occipital |
| Patient 11 | Oxford | 12y | M | Uncontrollable myoclonic epilepsy | Unknown | 32y | Cerebellum, occipital |
| Patient 12 | Oxford | 14y | F | Unknown | 48 | 17y | Cerebellum, occipital |
| Patient 13 | NBTR | 18y | F | Cardiac arrest | 24 | 2m | Cerebellum |
| Control 1 | NBTR | 44 d | F | Premature rupture of membranes with signs of neuromuscular disease. | 4m | 24h | Cerebellum |
| Control 2 | NBTR | 12m | N/A | Complications of bypass surgery | N/A | N/A | Cerebellum, occipital |
| Control 3 | NBTR | 13m | F | Occipital porencephaly | 2-4w | 72h | Cerebellum |
| Control 4 | NBTR | 2y | M | Unknown | 25y | Unknown | Cerebellum |
| Control 5 | Edinburgh | 16y | M | Suspension by ligature | 7d | 47h | Cerebellum, occipital |
| Control 6 | NBTR | 19y | F | Overdose | 18m | 5h | Cerebellum |
| Control 7 | Edinburgh | 22y | F | Poisoning | 7d | 44h | Cerebellum, occipital |
| Control 8 | Edinburgh | 24y | F | Suspension by ligature | 7d | 47h | Cerebellum, occipital |

| **Brain Region** | **Neuronal target** | **Respiratory chain targets** | **Primary Antibodies** | **Amplification** | **Secondary Antibodies** |
| --- | --- | --- | --- | --- | --- |
| **Occipital cortex** | Pyramidal neurons (SMI-32P, Covance) | Complex I | Mouse monoclonal **NDUFA13** (Abcam Ab110240; diluted 1:100)  Mouse monoclonal **COX4I2** (Abcam Ab110261; diluted 1:200)  Mouse monoclonal **SMI-32P** (Covance 801701; diluted 1:800) | Goat anti-mouse IgG2b biotin (Jackson Immunoresearch 115-005-207; diluted 1:100). | Hoescht  Goat anti-mouse IgG2b 488  Goat anti-mouse IgG2a 546  Goat anti-mouse IgG1 647 |
|  |  | Complex IV | Mouse monoclonal **COX1** (Abcam Ab14705; diluted 1:200)  Mouse monoclonal **Porin** (Abcam Ab14734; diluted 1:200)  Mouse monoclonal **SMI-32P** (Covance 801701; diluted 1:800) | None required. | Hoescht  Goat anti-mouse IgG2a 488  Goat anti-mouse IgG2b 546  Goat anti-mouse IgG1 647 |
|  | Interneurons (GAD65-67, Sigma) | Complex I & IV | Mouse monoclonal **NDUFB8** (Abcam Ab110242; diluted 1:100)  Mouse monoclonal **COX1** (Abcam Ab14705; diluted 1:200)  Mouse monoclonal **Porin** (Abcam Ab14734; diluted 1:200)  Rabbit polyclonal **GAD65-67** (Sigma; diluted 1:500) | Goat anti-mouse IgG1 biotin (Life technologies A10519; diluted 1:100). | Goat anti-mouse IgG1 546  Goat anti-mouse IgG2a 488  Goat anti-mouse IgG2b 647  Goat anti-rabbit IgG 405 |
| **Cerebellum** | Purkinje cells | Complex I & IV | Mouse monoclonal **NDUFB8** (Abcam Ab110242; diluted 1:100)  Mouse monoclonal **COX1** (Abcam Ab14705; diluted 1:200)  Mouse monoclonal **Porin** (Abcam Ab14734; diluted 1:200) | Goat anti-mouse IgG1 biotin (Life technologies A10519; diluted 1:100). | Hoescht  Goat anti-mouse IgG1 488  Goat anti-mouse IgG2a 647  Goat anti-mouse IgG2b 546 |

**Supplementary Table 2 – Primary and secondary antibodies used in this study.**
